# Reconstruction of a 2‑year neglected chronic patellar tendon rupture using peroneus longus autograft with suture anchor and cerclage wire augmentation: A case report and technical note

**DOI:** 10.1097/MD.0000000000050545

**Published:** 2026-09-04

**Authors:** Mao-Yi Yang, Li-Wei Hung

**Affiliations:** a Department of Orthopedic Surgery, Shin Kong Wu Ho-Su Memorial Hospital, Taipei, Taiwan; b School of Medicine, College of Medicine, Fu Jen Catholic University, New Taipei, Taiwan.

**Keywords:** autograft reconstruction, case report, neglected tendon rupture, patellar tendon rupture, peroneal longus tendon

## Abstract

**Rationale:**

Chronic patellar tendon rupture with complete tendon loss is difficult to reconstruct when primary repair is impossible. We report reconstruction using an ipsilateral peroneus longus (PL) autograft in a 4-strand “M-shaped” configuration with suture-anchor fixation and temporary cerclage augmentation.

**Patient concerns:**

A 17-year-old male had progressive anterior knee pain, instability, and inability to actively extend the left knee for 2 years after trauma.

**Diagnoses:**

Examination showed patella alta, a palpable infrapatellar gap, and complete loss of active extension. Radiographs showed an Insall–Salvati ratio of 1.28. Magnetic resonance imaging confirmed chronic complete patellar tendon rupture without a repairable stump.

**Interventions:**

A 21-cm ipsilateral PL autograft was folded into a 4-strand construct and anchored to the inferior patella and tibial tubercle. Patellar height was deliberately overcorrected, and a transpatellar–tibial cerclage wire protected the reconstruction during staged rehabilitation.

**Outcomes:**

At 1 year, after wire removal, the Insall–Salvati ratio was 1.08. The patient regained full active extension without lag, near-full flexion, and recreational activity participation without pain or donor-site morbidity. Asymptomatic wire breakage occurred at 6 months.

**Lessons:**

A multi-strand PL autograft with anchor fixation and temporary cerclage augmentation may restore patellar height and extensor function in chronic patellar tendon rupture with complete tendon loss.

## 1. Introduction

Patellar tendon rupture is an uncommon injury that disrupts the extensor mechanism of the knee, resulting in significant functional impairment and inability to perform daily activities.^[1]^ These injuries typically occur in males aged 30 to 40 years and are often associated with predisposing factors such as chronic tendinopathy, corticosteroid use, systemic diseases, or previous knee surgery.^[2,3]^ When diagnosed acutely, primary end-to-end repair with augmentation yields favorable outcomes.^[3]^ However, chronic or neglected ruptures present a considerable reconstructive challenge due to tendon retraction, scar tissue formation, quadriceps atrophy, and loss of viable tendon tissue for primary repair.^[4,5]^ The management of chronic patellar tendon ruptures remains controversial, with no universally accepted gold standard treatment.^[6]^

Various reconstruction techniques have been described in the literature, including autografts such as contralateral bone-patellar tendon-bone graft,^[5]^ ipsilateral hamstring tendons (semitendinosus and gracilis),^[4]^ quadriceps tendon turndown flaps,^[7]^ achilles tendon allograft,^[8,9]^ and synthetic materials including the ligament augmentation reconstruction system.^[10]^ Each approach has specific advantages and limitations regarding donor-site morbidity, graft strength, incorporation time, and technical complexity. The peroneal longus tendon has emerged as a valuable autograft source in various foot and ankle reconstructive procedures due to its favorable biomechanical properties, sufficient length, and relatively low donor-site morbidity.^[11,12]^ However, its use for patellar tendon reconstruction has been rarely reported in the orthopedic literature. The peroneal longus tendon offers several theoretical advantages including ipsilateral harvest, avoidance of knee joint violation, adequate tensile strength, and preservation of other commonly used grafts for potential revision procedures.

We present a case of successful reconstruction of a 2-year-old neglected patellar tendon rupture in a 17-year-old male using ipsilateral peroneal longus tendon autograft with suture anchor fixation, augmented by cerclage wiring. To our knowledge, this represents one of the few reported cases utilizing the peroneal longus tendon for patellar tendon reconstruction, demonstrating that this technique can achieve satisfactory functional recovery with acceptable donor-site morbidity. This case adds to the limited literature on reconstructive options for chronic patellar tendon ruptures and highlights the versatility of the peroneal longus tendon as an autograft source in complex knee ligament reconstruction.

## 2. Case presentation

A 17-year-old male presented to our orthopedic clinic with a 2-year history of progressive left knee pain, instability, and complete inability to extend the left knee. The patient reported sustaining a fall at age 15 while chasing a bus, after which he experienced immediate anterior knee pain and difficulty walking. Despite seeking initial medical attention at a local clinic, the diagnosis of patellar tendon rupture was missed, and the patient was treated conservatively with immobilization and analgesics. Over the subsequent 2 years, the patient experienced progressive functional deterioration with persistent quadriceps weakness and difficulty rising from a seated position. The patient denied any history of prior knee injuries, surgeries, corticosteroid injections, or systemic diseases.

On physical examination, the patient demonstrated a significant antalgic gait with a marked quadriceps avoidance pattern. Inspection revealed mild knee effusion and absence of a palpable patellar tendon. The patella demonstrated superior positioning compared to the contralateral knee, with a palpable defect between the inferior pole of the patella and the tibial tubercle. Active knee extension was completely absent, with the patient unable to perform a straight leg raise against gravity. However, the patient retained full passive range of motion from 0 to 135 degrees of flexion. With the knee in 90 degrees of flexion, a visible gap was observed below the kneecap, indicating loss of extensor mechanism integrity.

**Figure 1.**
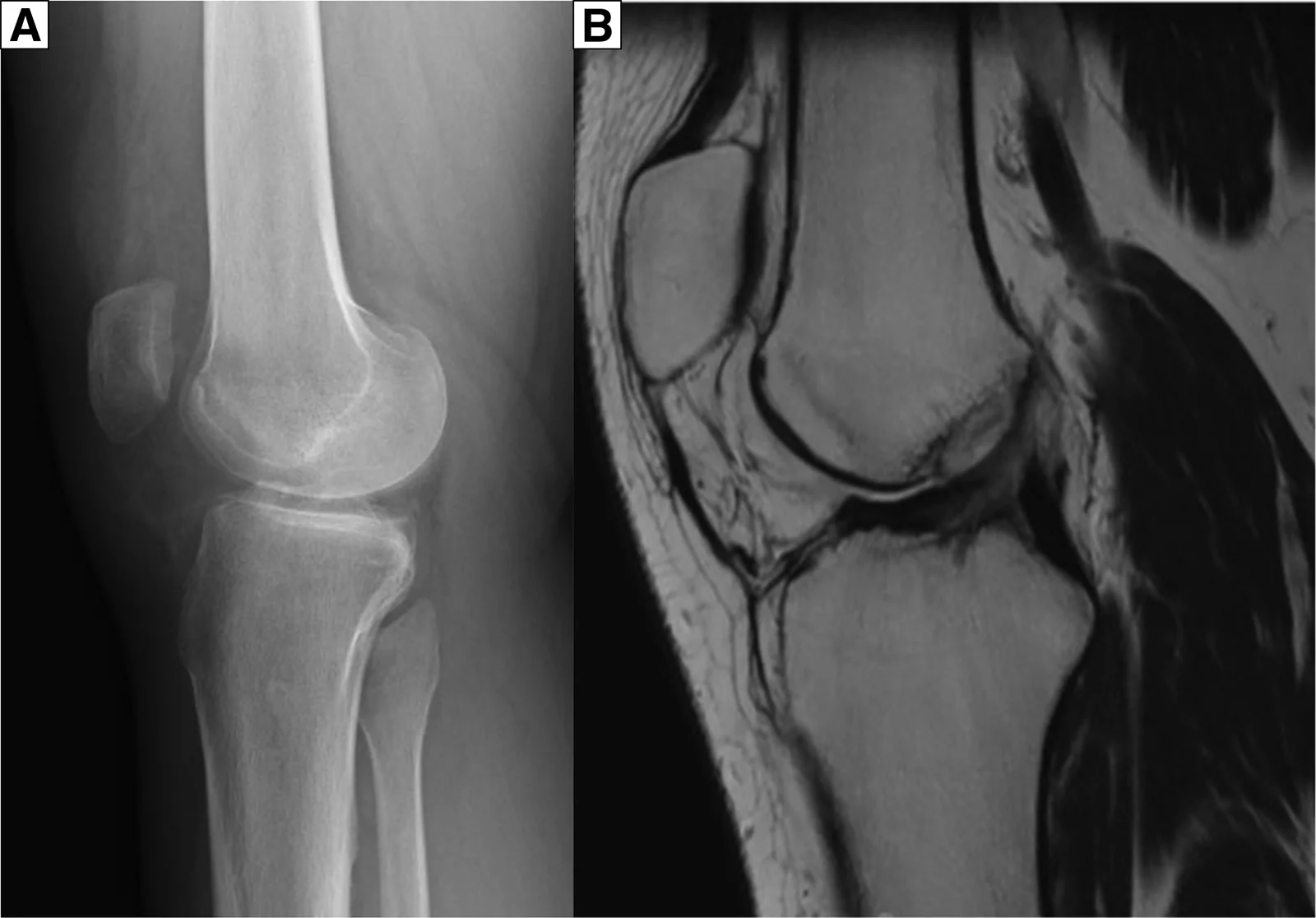
Preoperative imaging of the left knee. (A) Lateral radiograph demonstrating patella alta with an Insall–Salvati ratio of 1.28. (B) Sagittal magnetic resonance image confirming complete disruption of the patellar tendon.

Plain radiographs of the left knee demonstrated superior migration of the patella (patella alta) with an abnormal Insall–Salvati ratio of 1.28 (normal 0.8–1.2), confirming chronic patellar tendon disruption (Fig. 1). No evidence of patellar fracture, avulsion fragments, or degenerative changes was noted. Magnetic resonance imaging of the left knee confirmed complete disruption of the patellar tendon with scar tissue filling the defect between the patella and tibial tubercle (Fig. 1). Based on these clinical and radiological findings, the diagnosis of chronic neglected complete patellar tendon rupture was established, and surgical reconstruction was planned.

The patient underwent surgical reconstruction under spinal anesthesia in the supine position with a thigh tourniquet. A longitudinal midline incision was made over the anterior aspect of the left knee, extending from the top of the patella to the tibial tubercle. Careful dissection was performed through subcutaneous tissue, revealing extensive scar tissue and granulation tissue at the site of the patellar tendon rupture. The proximal and distal remnants of the patellar tendon were identified, debrided, and excised, as the tissue quality was inadequate for reconstruction. The inferior pole of the patella and the tibial tubercle attachment site were prepared by removing scar tissue and creating a raw bony surface to promote biological healing.

A separate incision was made over the lateral aspect of the ipsilateral left leg to harvest the peroneal longus tendon. The tendon was carefully dissected free, preserving the peroneus brevis tendon to maintain eversion function. An adequate length of tendon, approximately 21 cm, was harvested. The tendon was prepared on the back table by folding it to create a 4-strand construct for increased mechanical strength.

**Figure 2.**
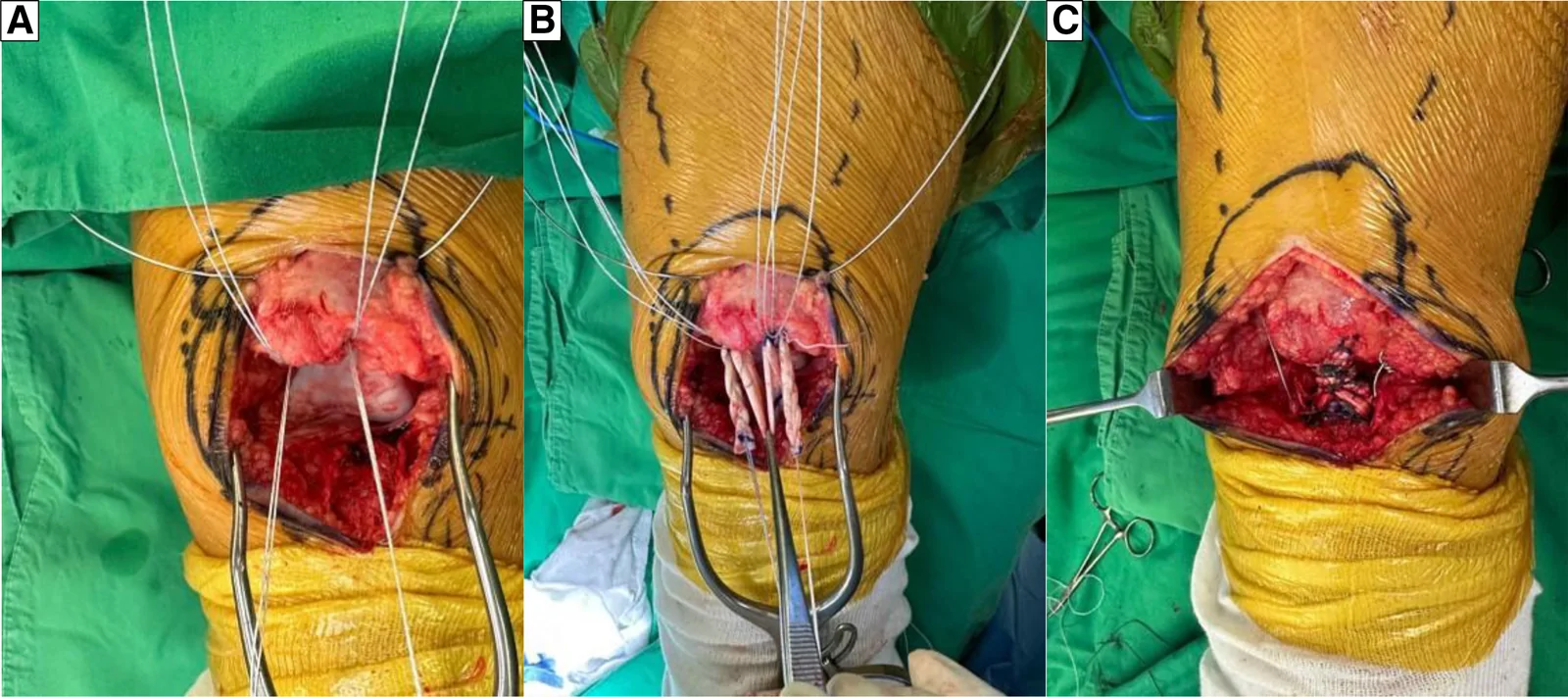
Intraoperative surgical technique. (A) The inferior patellar pole and tibial tubercle were debrided to create a suitable bed for suture anchor insertion. (B) The peroneus longus tendon autograft was configured in an “M-shaped” fashion for patellar tendon reconstruction. (C) High-strength (Hi-Fi) sutures were placed in the midportion of the peroneus longus graft, and a cerclage wire loop was added for additional augmentation.

Patellar tendon reconstruction was performed using a 4-stranded peroneus longus tendon (PL) graft arranged in an “M-shaped” configuration. Two double-loaded Q-Fix suture anchors (Smith & Nephew) were placed into the inferior pole of the patella and 2 at the tibial tubercle, serving as insertion sites for the graft. The PL graft was folded evenly to form 4 limbs: the 2 central limbs (the peaks of the “M”) were anchored into the inferior pole of the patella, while the 2 lateral limbs were anchored into the tibial tubercle (Fig. 2).

**Figure 3.**
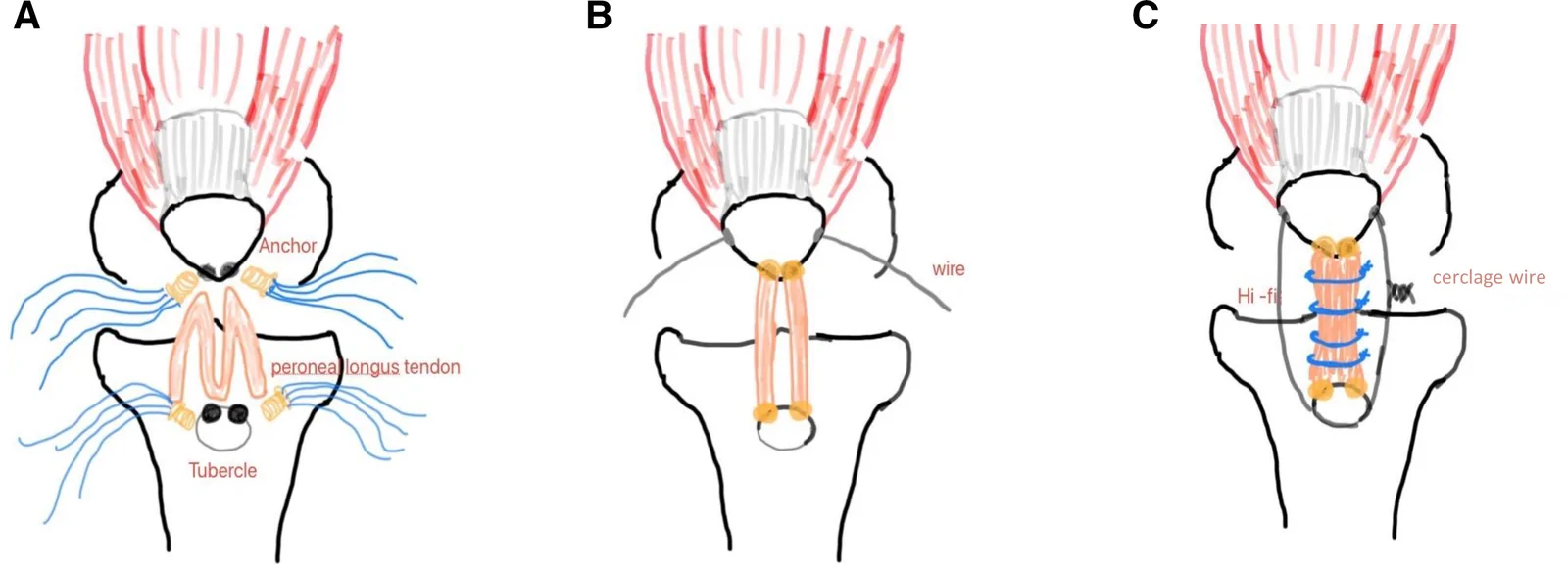
Schematic illustration of the surgical technique. Stepwise technique of patellar tendon reconstruction using a peroneus longus autograft: (A) The autograft is configured in an M-shaped geometry between the inferior pole of the patella and the tibial tubercle and secured with suture anchors. (B) Placement of a trans-patellar–tibial protective cerclage wire provides tension-band reinforcement. (C) The final construct demonstrating high-strength sutures securing the midportion of the graft and the tightened cerclage wire providing load-sharing protection over the reconstructed patellar tendon.

To counteract chronicity-related quadriceps contracture and accommodate anticipated secondary graft stretch (creep effect) during postoperative rehabilitation, graft tensioning was performed with the knee in full extension (0 degree). Under maximal manual traction, the patella was drawn inferiorly prior to final anchor fixation, achieving an immediate postoperative Insall–Salvati ratio of 0.80 on postoperative day 0. Hi-Fi high-strength sutures were utilized to secure and bundle the midsegment of the folded graft, creating a unified reconstruction. For additional protection and load-sharing during the early rehabilitation period, a 1.2 mm Kirschner wire was passed through the patella and tibial tubercle, forming a cerclage construct that was tightened for reinforcement. This overall technique allowed for precise structural reconstruction of the extensor mechanism, providing adequate primary stability while allowing progressive restoration of balanced patellar height throughout the knee’s range of motion. (See Fig. 3 for a schematic illustration of the surgical technique.)

Postoperatively, the knee was immobilized in extension in a hinged brace for 4 weeks, with toe-touch weight-bearing allowed and basic quadriceps activation exercises initiated under protection. Range of motion and weight-bearing were then gradually progressed, and more intensive active extension and strengthening exercises were started at approximately 3 months once the reconstruction was deemed sufficiently healed and the patient entered formal supervised physical therapy.

**Figure 4.**
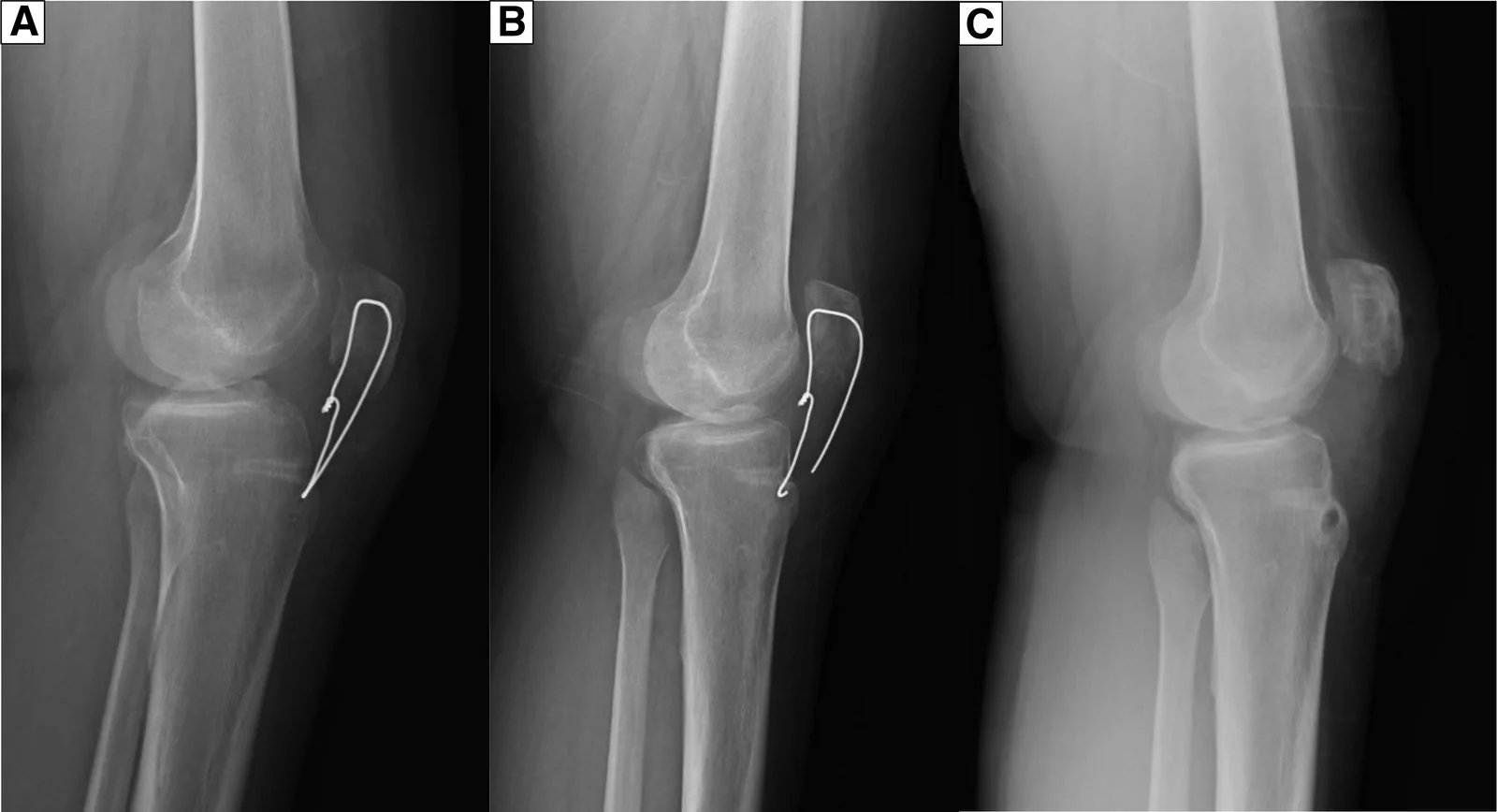
Serial postoperative lateral radiographs. (A) Immediate postoperative lateral radiograph (postoperative day 0) demonstrating an Insall–Salvati ratio of 0.80 following intraoperative tensioning. (B) Radiograph obtained 6 months postoperatively incidentally demonstrating breakage of the cerclage wire. (C) Radiograph obtained 12 months postoperatively after hardware removal demonstrating maintained patellar height with a balanced Insall–Salvati ratio of 1.08.

At 6 months postoperatively, lateral radiographs incidentally demonstrated cerclage wire breakage, but the patient remained asymptomatic with no anterior knee pain, instability, or functional limitations, and had achieved active range of motion from 0 to 120 degrees with return to usual daily activities (Fig. 4).

At 1-year follow-up, the cerclage wire was removed. Post-removal radiographs showed maintained patellar height with an Insall–Salvati ratio of 1.08 (Fig. 4). Clinical examination revealed full active knee extension without extensor lag, active range of motion from 0 to 130 degrees, no patellofemoral crepitus, a negative patellar apprehension test, and unrestricted participation in recreational activities.

**Figure 5.**
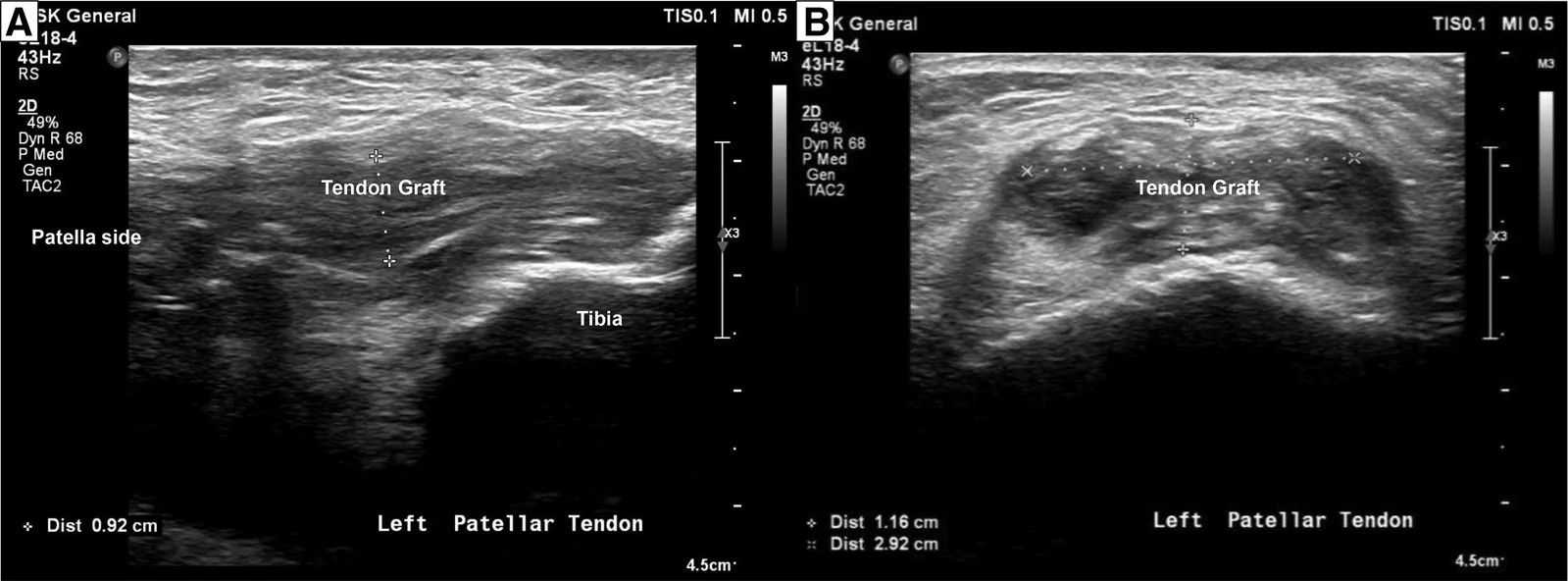
One-year postoperative ultrasonography of the left knee. (A) Longitudinal view along the long axis of the extensor mechanism demonstrating structural continuity of the reconstructed peroneus longus autograft from the patellar insertion to the tibial attachment. (B) Transverse view at the mid-substance level showing a well-organized, thick graft construct with preserved structural integrity and no evidence of intratendinous disruption.

Ultrasonography at 1 year (Fig. 5) demonstrated an intact peroneus longus tendon graft. The longitudinal view (Fig. 5A) confirmed continuous structural integrity of the graft from the patellar insertion side to the tibial side, while the transverse view (Fig. 5B) demonstrated a well-organized, thick graft construct without evidence of attenuation or defect. Clinical evaluation at 1 year revealed that the patient experienced no anterior knee pain, had no limitations in daily activities, and successfully returned to recreational activities including cycling and swimming. Importantly, no donor-site morbidity was reported at the peroneal tendon harvest site, with maintained ankle eversion strength and no subjective ankle instability. Overall, the patient expressed high satisfaction with the final functional outcome.

## 3. Discussion

Chronic patellar tendon rupture represents a challenging clinical scenario that requires complex reconstruction when primary repair is not feasible because of tendon loss, retraction, and poor tissue quality.^[13,14]^ In this case, an ipsilateral PL tendon autograft combined with suture anchor fixation and cerclage wire augmentation successfully restored extensor mechanism function in a 2‑year neglected rupture, achieving satisfactory functional outcomes with minimal donor-site morbidity.

Multiple reconstructive options have been described for chronic patellar tendon rupture, including contralateral bone-patellar tendon-bone autograft, ipsilateral hamstring tendons, Achilles tendon allograft, and synthetic ligaments such as ligament augmentation reconstruction system.^[15,16]^ Each technique carries trade-offs in terms of graft strength, incorporation, donor-site morbidity, and technical complexity. Contralateral bone-patellar tendon-bone graft offers robust bone-to-bone healing but sacrifices a healthy tendon and exposes the contralateral knee to risks such as fracture and anterior knee pain.^[17]^ Ipsilateral hamstring autografts avoid contralateral morbidity yet may provide insufficient length and strength for large defects.^[18–20]^ Allograft solutions provide adequate length but depend on tissue banking and and may have delayed incorporation and a small risk of disease transmission,^[21,22]^ whereas synthetic ligaments provide immediate strength but lack biological incorporation and raise concerns regarding infection and long-term durability.^[23,24]^

The PL tendon is a well-established autograft source in foot and ankle reconstruction but remains underutilized for extensor mechanism reconstruction.^[12,25,26]^ It offers several advantages for chronic patellar tendon rupture: it typically provides 20 to 30 cm of graft length, allowing multi-strand folding to increase mechanical strength^[27]^; its tensile properties are comparable to other commonly used autografts^[28]^; and ipsilateral harvest preserves the contralateral limb and other potential grafts for future reconstruction if needed. Donor-site morbidity is generally low when the peroneus brevis tendon is preserved, as it is the dominant evertor, and clinical studies have reported minimal loss of ankle eversion strength or subjective instability.^[29–31]^ Consistent with these reports, our patient experienced no donor-site pain or ankle instability at 1 year.

Fixation strategy is critical for balancing mechanical stability and bone preservation. Traditional transosseous tunnel techniques – such as those utilizing bone tunnels through the patella and tibial tuberosity – offer favorable tendon-to-bone healing potential within osseous tunnels.^[32]^ However, transosseous drilling in compromised, chronically retracted, or osteopenic bone carries an inherent risk of iatrogenic patellar fractures, tunnel convergence, or anterior cortical breach during tensioning.^[33,34]^ In contrast, suture anchor fixation with an onlay graft arrangement provides a reproducible, low-profile alternative that avoids extensive bony disruption.^[35]^ While onlay reconstruction relies primarily on surface interface healing, the broad contact area provided by the M-shaped multi-strand configuration, combined with rigid anchor fixation and protective cerclage wiring, mitigates interface movement and supports robust biological integration without compromising patellar structural integrity.^[36]^

Several technical principles were applied to address the chronicity of the rupture. Thorough debridement of scar tissue and preparation of healthy bone at the inferior patellar pole and tibial tubercle were performed to optimize graft incorporation. Folding the PL tendon into a multi-strand construct increased initial mechanical strength, which is particularly important before complete biological healing. Because tendon grafts behave as viscoelastic tissues and may undergo early creep and irreversible elongation under cyclic loading,^[37–39]^ intentional intraoperative tensioning was performed with the knee in full extension and the patella slightly overcorrected, yielding an immediate postoperative Insall–Salvati ratio of 0.80. This controlled overcorrection anticipated secondary biological relaxation during rehabilitation and ultimately resulted in a balanced Insall–Salvati ratio of 1.08 at 1 year. Temporary cerclage wire augmentation provided additional load-sharing protection during the early healing phase, reducing stress on the biological graft and potentially lowering the risk of re-rupture.^[40]^

The cerclage wire breakage observed at 6 months occurred after the critical early healing window, when the graft had likely achieved sufficient intrinsic strength. The patient remained asymptomatic with preserved patellar height and stable knee function, supporting the concept that the cerclage construct served as temporary protection rather than a long-term load-bearing component. The mild residual patella alta noted at final follow-up represented a substantial improvement from the preoperative state and falls within a range generally considered compatible with good functional outcomes after chronic patellar tendon reconstruction.^[41]^

This report has several limitations. As a single case, it cannot establish comparative superiority over other reconstructive techniques or account for the influence of patient age, chronicity, and individual biology on outcome. The 1-year follow-up, although adequate to demonstrate early graft incorporation and functional recovery, does not capture long-term durability, re-rupture risk, or potential late patellofemoral degenerative changes. Furthermore, the individual contributions of the multi-strand PL configuration, suture anchor fixation, and cerclage wire augmentation cannot be isolated.

Despite these limitations, this case suggests that PL autograft reconstruction with multi-strand anchor-based fixation and temporary cerclage augmentation is a viable and effective option for complex, neglected patellar tendon ruptures. The technique restored extensor mechanism function, maintained near-anatomic patellar height, and preserved donor-site function in a challenging 2-year neglected rupture. Larger series and comparative studies are needed to further define the long-term efficacy and optimal indications of this approach relative to established reconstructive strategies.

## 4. Conclusion

Chronic patellar tendon rupture with complete tendon loss can be effectively reconstructed using a peroneus longus autograft with suture anchor fixation and cerclage wire augmentation. This technique provides primary stability, restores active knee extension with near-full range of motion, and results in minimal donor-site morbidity. Peroneus longus autograft represents a versatile, highly valuable alternative for complex extensor mechanism reconstruction, particularly when preserving other autograft options is desired or contralateral harvest is unfeasible.

## Author contributions

**Conceptualization:** Li-Wei Hung.

**Data curation:** Mao-Yi Yang.

**Methodology:** Mao-Yi Yang.

**Supervision:** Li-Wei Hung.

**Validation:** Mao-Yi Yang.

**Visualization:** Mao-Yi Yang.

**Writing – original draft:** Mao-Yi Yang.

**Writing – review & editing:** Mao-Yi Yang.
